# Pulmonary Vein Balloon Angioplasty and Stenting for Left Superior and Inferior Pulmonary Vein Stenosis/Occlusion After Radiofrequency Ablation for Paroxysmal Atrial Fibrillation

**DOI:** 10.1002/ccr3.70325

**Published:** 2025-03-24

**Authors:** Wen Pan, Jianfeng Qian, Licheng Lu, Haixiang Xu, Jianhua Fan

**Affiliations:** ^1^ Department of Cardiology Kunshan Hospital of Traditional Chinese Medicine Suzhou Jiangsu China

**Keywords:** atrial fibrillation, case report, pulmonary hypertension, pulmonary vein balloon angioplasty or stent implantation, pulmonary vein stenosis or occlusion, radiofrequency ablation

## Abstract

Early detection and prompt intervention are essential for managing pulmonary vein stenosis and occlusion (PVS/O) following radiofrequency ablation (RFA) for atrial fibrillation (AF). This case report highlights the importance of considering PVS/O in patients with dyspnea and cough post‐RFA and underscores the effectiveness of balloon angioplasty and stent implantation in alleviating symptoms and reducing the risk of pulmonary hypertension.

## 1 Introduction

Radiofrequency ablation (RFA) is a frequently employed method for managing paroxysmal atrial fibrillation (AF). However, complications might arise, including pulmonary vein (PV) stenosis and occlusion (PVS/O) that necessitate immediate recognition and intervention.

We report a case of a female patient with dyspnea and cough 6 months after her third RFA. The patient was diagnosed with left superior PV (LSPV) occlusion and left inferior PV (LIPV) stenosis and was successfully treated with PV balloon angioplasty and stent implantation.

## 2 Case History

**FIGURE 1:**
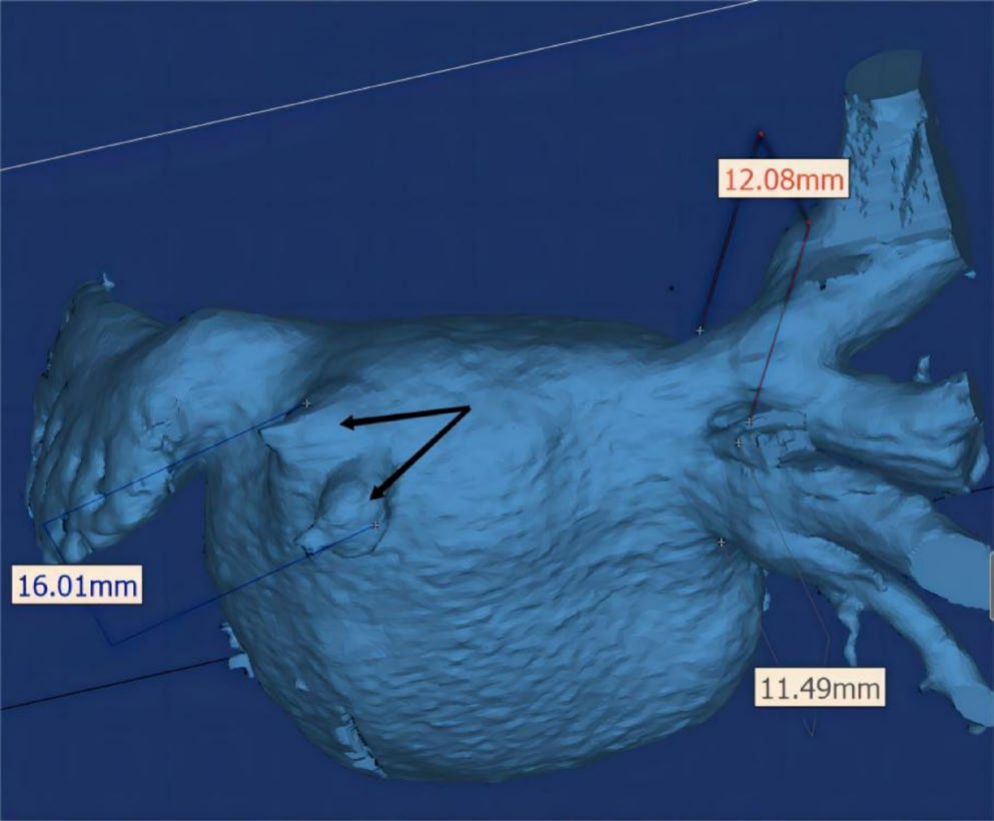
CT scan showing total occlusion of the left superior pulmonary vein and sub‐occlusion of the left inferior pulmonary vein (arrows).

A 75‐year‐old woman presented with the onset of progressive dyspnea on exertion and a nonproductive cough 6 months after her third RFA for Paroxysmal AF. Transthoracic echocardiography indicated a pulmonary arterial pressure of 69 mmHg and enlargement of the right ventricle and atrium. The treatment for right heart failure was ineffective. A computed tomography (CT) angiogram revealed LSPV occlusion and LIPV subtotal occlusion (Figure 1). The patient was subsequently referred to cardiac catheterization.

## 3 Investigations and Treatment

Cardiac catheterization revealed pulmonary arterial hypertension of 54/17 (32) mmHg. A pulmonary artery wedge angiography was performed, with an angiographic catheter positioned in the superior/inferior branch of the left pulmonary artery to visualize the anatomy of the affected PV and locate its ostium.

Access to the PVs was achieved via a transseptal approach using a Brockenbrough needle and an 8.5F long sheath. A 5F multipurpose catheter was employed to engage the left atrium and selectively navigate to the ostia of the LSPV and LIPV under fluoroscopic guidance. Angiograms of the left PVs revealed 90% stenosis of the LIPV and total occlusion of the LSPV. The occlusion in the LSPV was located at the ostium, with complete loss of blood flow distal to the occlusion. For the LIPV, stenosis was para‐ostial, with a visible residual lumen.

Recanalization of the LSPV was achieved using a 0.018″ V‐18 control wire (Boston Scientific), which was advanced under fluoroscopic guidance with the support of a 5F multipurpose catheter to maintain luminal positioning (Video 1).

**VIDEO 1:** Recanalization of the LSPV. In this video, the recanalization process of the LSPV is demonstrated. Initially, a 0.018" V—18 control wire (Boston Scientific) is introduced. Under fluoroscopic guidance, with the support of a 5F multipurpose catheter, the wire is carefully advanced toward the occluded segment of the LSPV. As the wire progresses, it can be seen gradually navigating through the occluded area. The 5F multipurpose catheter plays a crucial role in maintaining the position of the wire within the lumen, ensuring stable advancement. This process is meticulous, with the operator closely monitoring the position of the wire on the fluoroscopic screen to avoid any potential damage to the vessel wall. Video content can be viewed at https://onlinelibrary.wiley.com/doi/10.1002/ccr3.70325

**FIGURE 2:**
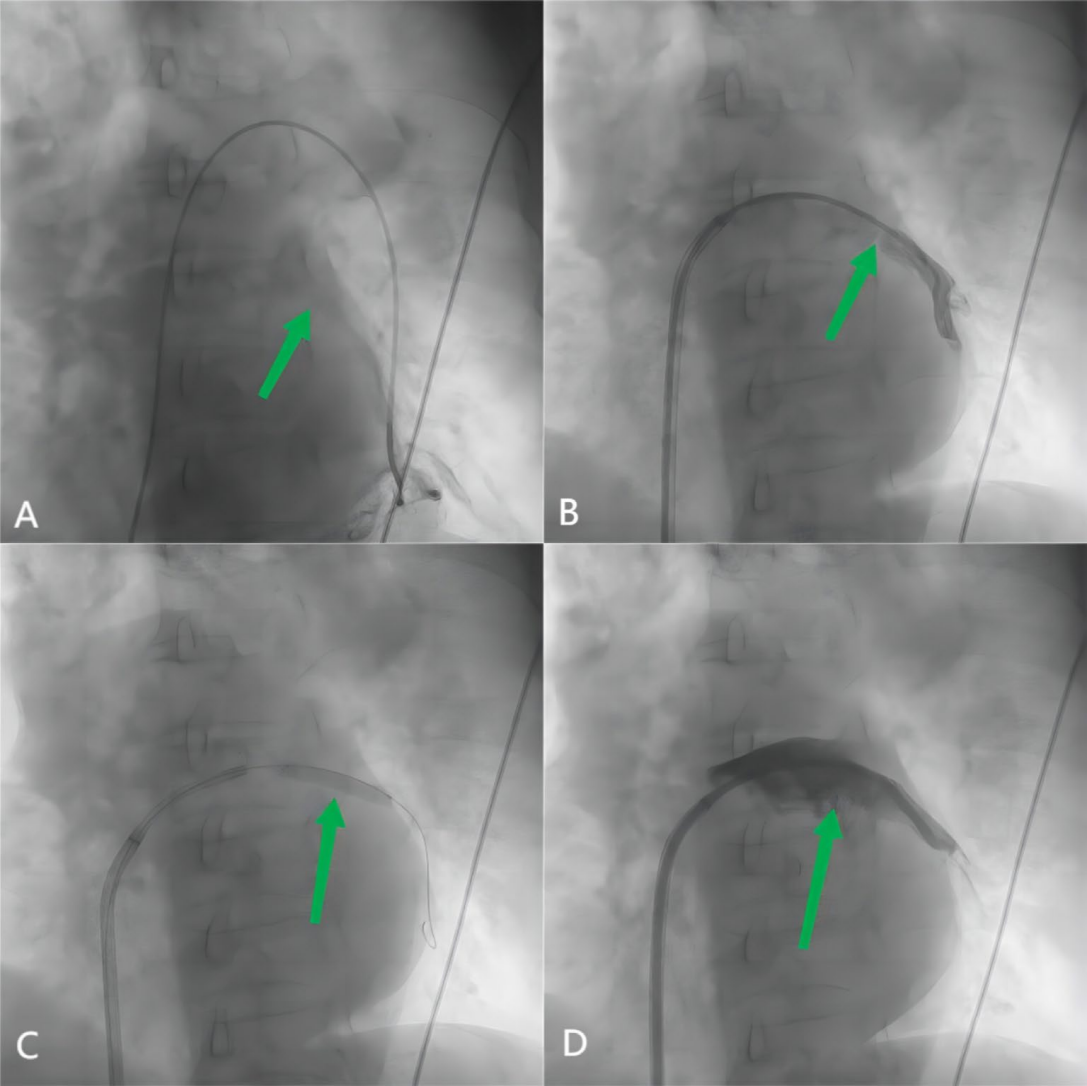
(A) Left inferior pulmonary arterial wedge angiogram defined the pulmonary vein anatomy to guide the intervention(arrow). (B) Angiography showing sub‐occlusion of the left inferior pulmonary vein (arrow). (C) A 5‐mm‐diameter balloon is inflated within the left inferior pulmonary vein ostia(arrow). (D) Angiography showing a stent implanted in the inferior pulmonary vein with a good result (arrow).

**FIGURE 3:**
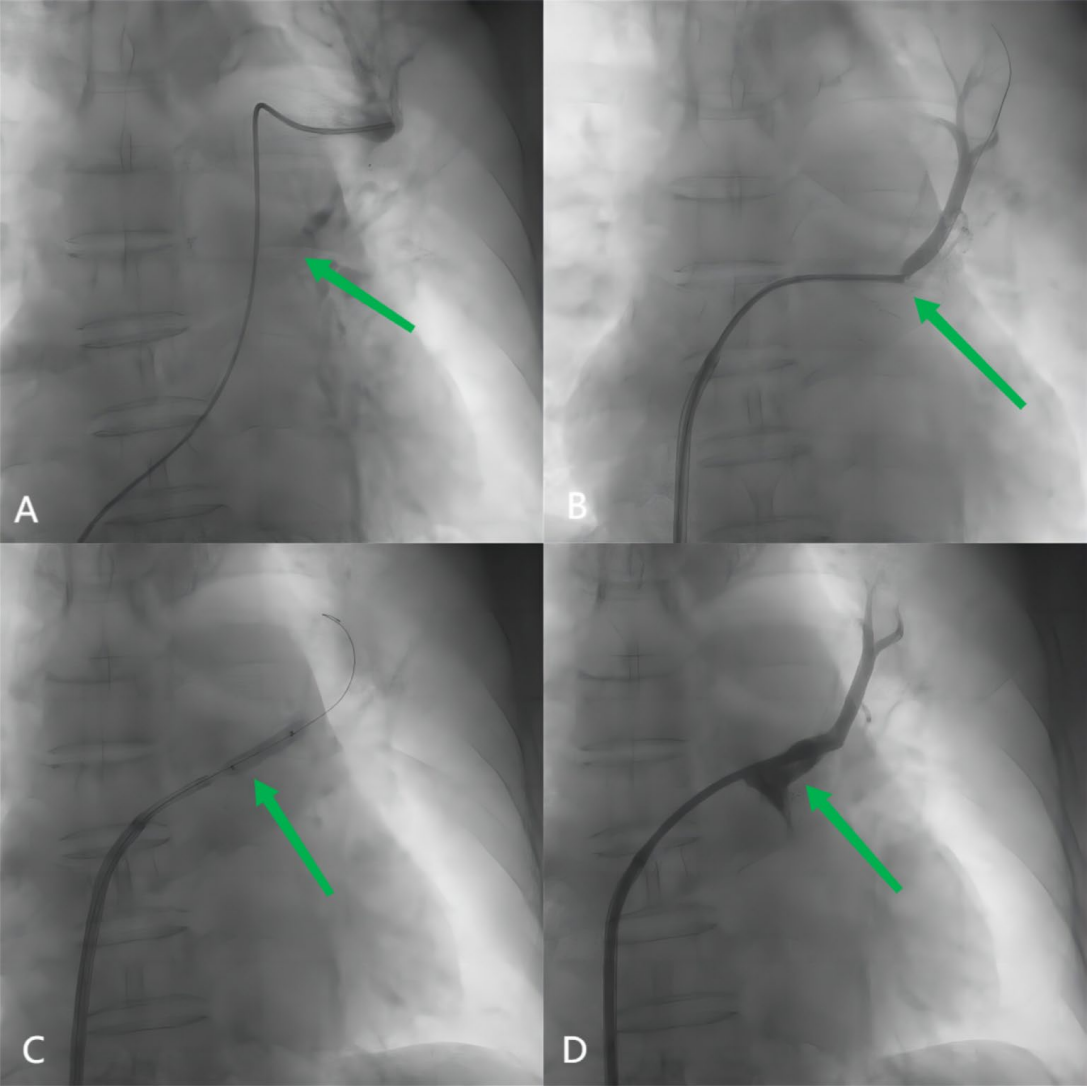
(A) Left superior pulmonary arterial wedge angiogram defined the pulmonary vein anatomy to guide the intervention (arrow). (B) Angiography showing total occlusion of the left superior pulmonary vein (arrow). (C) A 4‐mm‐diameter balloon is inflated within the left superior pulmonary vein ostia (arrow). (D) Angiography showing a stent implanted in the superior pulmonary vein with good results (arrow).

Sequential balloon dilations were performed using a 2.0 mm balloon at 8 atm for 10 s and a 4.0 mm balloon at 10 atm for 15 s to create an initial lumen. Pre‐dilation was performed with a 5.0 × 20 mm Armada balloon at 12 atm for 15 s to optimize the occlusion for stenting. An 8.0 × 19 mm Omnilink Elite bare metal stent was deployed at 16 atm for 30 s, followed by post‐dilation at the same pressure and duration to ensure proper expansion and apposition. For the LIPV, balloon angioplasty was performed with a 5.0 × 30 mm balloon at 12 atm for 15 s. Stenting was performed with a 10.0 × 19 mm Omnilink Elite bare metal stent deployed at 14 atm for 20 s, with post‐dilation at the same pressure and duration to achieve optimal results. Balloon and stent sizes were selected based on pre‐procedural imaging, including CT angiography and angiographic measurements. These interventions achieved excellent angiographic results for both veins (Figure 2 for LIPV and Figure 3 for LSPV). After the operation, the pulmonary arterial pressure reduced to 45/15 (27) mmHg.

## 4 Outcome and Follow‐Up

The day after the procedure, transthoracic echocardiography showed improved symptoms, with pulmonary arterial pressure reduced to 39 mmHg. The patient was prescribed 15 mg/day of rivaroxaban and 75 mg/day of clopidogrel hydrogen sulfate.

At 3 months post‐procedure, the patient demonstrated significant symptomatic improvement and echocardiographic evidence of reduced pulmonary hypertension. Specifically, the left atrial dimension decreased from 41 to 39 mm, the right atrial dimension from 51 to 42 mm, and the pulmonary artery pressure from 69 to 43 mmHg. However, the patient did not return for further follow‐up, likely due to the significant alleviation of symptoms, which may have reduced her perceived need for ongoing medical evaluation.

## 5 Discussion

PVS was most commonly observed in the LSPV, affecting 18 out of 54 patients (33%), followed by the LIPV, which affected 16 out of 54 patients (30%) [1]. PVO is defined as over 95% stenosis or complete loss of PV patency. About 23% of patients with PVS gradually progress to PVO [2].

Chronic PVS/O following RFA for AF can lead to lasting venous and arterial alterations within the lungs, affecting areas both proximal and distal to the ablation site [3]. Histological studies have indicated that these changes include intimal thickening with organized thrombus, necrotic myocardium in various stages of fibrosis, endovascular contraction, and proliferation of elastic lamina [4]. Pathological alterations persist beyond the immediate ablation area, impacting both intraPVs and arteries. These structural changes are both a result of and a contributor to the development of pulmonary hypertension (PH).

It is essential to recognize and manage these complications promptly to avoid progressive respiratory compromise and related complications. In the early stages, the PVS incidence after AF ablation reached 42.4% [5], but with the modification of ablation strategies, technological advancements, and reliable monitoring, this complication has decreased to approximately 0.36%–0.78% [1, 6].

Symptom severity is attributable to factors, including the number of veins affected, the presence of collateral, and the rate of progression. The clinical manifestations of PVS/O are quite variable, including dyspnea, cough, and hemoptysis, which mimic other common cardiac and pulmonic pathologies, making the disease a challenging clinical diagnosis [7]. The onset of PVS/O in these symptomatic patients varies, typically occurring between 3 and 6 months after the RFA procedure [8]. As the disease progresses, some patients may develop refractory heart failure and hemoptysis. Consequently, it is essential to consider the possibility of PVS/O for patients who develop symptoms after AF ablation.

Interventional treatment for patients with acquired PVS/O includes balloon angioplasty or stent implantation, while surgical treatment options, including sutureless venoplasty or pericardial patchplasty, are available for those unsuitable for interventional therapy. Multiple investigations have demonstrated that the incidence of restenosis is lower after stenting compared to balloon angioplasty [7, 9], and a peripheral large‐diameter bare metal stent is preferable to a coronary drug‐eluting stent due to its lower restenosis rate [10]. In addition to in‐stent restenosis, potential complications of percutaneous PV interventions include PV rupture, stroke, and phrenic nerve injury; however, these are exceptionally rare events [11].

This case underscores several important considerations in the management of PVS/O following RFA. The complexity of diagnosing and treating PVS/O emphasizes the need for careful clinical evaluation, advanced imaging, and meticulous procedural techniques.

The diagnosis of PVS/O in this patient was challenging due to the nonspecific nature of the symptoms, which included dyspnea and cough, commonly observed in conditions such as chronic obstructive pulmonary disease or right heart failure. These overlapping clinical features delayed the recognition of PVS/O, highlighting the importance of maintaining a high index of suspicion in patients with unexplained respiratory symptoms after RFA. Advanced imaging modalities, particularly CT angiography and selective pulmonary angiography, were essential in confirming the diagnosis and guiding subsequent management.

The successful treatment of this patient was also complicated by the complete occlusion of LSPV, which required a carefully planned interventional strategy. Precise localization of the ostium and stepwise balloon dilation were critical to safely re‐establishing the lumen without causing vessel injury. The use of a 0.018″ V‐18 control wire provided excellent torque control, facilitating the recanalization process and ensuring accurate positioning of the balloons and stents. Sequential balloon dilation with incremental increases in balloon size and pressure is crucial to safely treat completely occluded veins, reducing the risk of complications. The deployment of large‐diameter bare metal stents was pivotal in achieving long‐term patency while minimizing the risk of restenosis. Real‐time angiographic imaging should be employed throughout the procedure to guide wire and balloon placement and to evaluate the procedural outcomes in real time.

Notably, the procedure was completed without any major complications, such as vessel rupture or thromboembolism, which highlights the importance of meticulous procedural planning, skillful execution, and close monitoring during the intervention. Although alternative strategies, such as using specialized guidewires with enhanced torque control or smaller‐diameter balloons for initial channel creation, were considered, they were not required given the success of the primary approach. Surgical interventions, including sutureless venoplasty, were reserved as a contingency for cases where percutaneous techniques might fail, further emphasizing the value of a tailored, stepwise approach to managing complex cases of PVS/O.

Despite significant clinical and echocardiographic improvements observed at 3 months post‐procedure, long‐term follow‐up remains essential. Continued monitoring is required to detect late complications, such as in‐stent restenosis or recurrent PVS/O, which may occur over time. We will make efforts to contact the patient for additional follow‐up evaluations. While the lack of immediate complications in this case is promising, further data from extended follow‐up will be invaluable in assessing the durability and long‐term safety of these interventional strategies.

## 6 Conclusion

This case highlights the development of PH and symptoms 6 months after a third RFA for paroxysmal AF. The patient's symptoms and PH resolved following stenting of the LSPV and LIPV. PVS/O should be considered in patients presenting with dyspnea and nonproductive cough after catheter‐based RFA for AF. Timely diagnosis and intervention are crucial for optimal treatment and outcomes.

## Author Contributions

**Wen Pan:** conceptualization, data curation, writing – original draft. **Jianfeng Qian:** conceptualization, supervision, visualization. **Licheng Lu:** conceptualization, supervision, visualization. **Haixiang Xu:** writing – original draft. **Jianhua Fan:** conceptualization, project administration, supervision, writing – review and editing.

## Disclosure

The authors have nothing to report.

## Ethics Statement

This study received ethical approval from the Ethics Committee of Kunshan Hospital of Traditional Chinese Medicine.

## Consent

Written informed consent was obtained from the patient to publish this case report in accordance with the journal's patient consent policy.

## Data Availability

The data that support the findings of this study are available upon request from the corresponding author.
